# Abdominal and Pelvic Computed Tomography for the Diagnosis of ST-Elevation Myocardial Infarction: The Challenges of Acute Coronary Syndrome in Diabetic Patients

**DOI:** 10.7759/cureus.30816

**Published:** 2022-10-28

**Authors:** Blake Briggs, Edan Zitelny, Tamir Zitelny

**Affiliations:** 1 Emergency Medicine, University of Tennessee Medical Center, Knoxville, TN, USA; 2 Internal Medicine, University of Pennsylvania Department of Medicine, Philadelphia, USA; 3 Emergency Medicine, Wake Forest School of Medicine, Winston-Salem, USA

**Keywords:** computed tomography, diabetic autonomic neuropathy, radiology, atypical chest pain, stemi

## Abstract

Diabetes mellitus (DM) is a major independent risk factor for cardiovascular disease. Patients who present with the metabolic emergency of diabetic ketoacidosis (DKA) have similar symptoms of diaphoresis, nausea, emesis, and abdominal pain, which can conceal acute coronary syndrome (ACS). We present a unique case where computed tomography (CT) of the abdomen and pelvis with IV contrast played an integral role in diagnosing an acute myocardial infarction in a patient with no typical ischemic symptoms.

A 56-year-old female presented to the emergency department with abdominal pain, nausea, and vomiting. She was suspected of having DKA. Aggressive management was started, including weight-based appropriate IV regular insulin. A CT abdomen and pelvis with IV contrast was performed due to persistent abdomen pain. This demonstrated severe hypoattenuation of the posteroinferior aspect of the left ventricular wall. An EKG was immediately performed and was consistent with an inferior STEMI. The patient was taken to the interventional cardiology suite where they found the culprit lesion to be mid-circumflex with 100% stenosis.

This case highlights many important lessons in approaching diabetic patients who are presenting with DKA. DM is associated with cardiac autonomic neuropathy (CAN), a condition that greatly influences perceived chest pain. While little is known about this condition, some manifestations include resting tachycardia, exercise intolerance, orthostatic hypotension, and an increased risk of silent myocardial infarction. Critically, providers must maintain a low threshold to assess for cardiac ischemia in diabetic patients and more readily obtain EKGs in triage as well as during the patient’s course in the ED to prevent complications from delayed ACS care.

## Introduction

Diabetes mellitus (DM) is a major independent risk factor for cardiovascular disease, with nearly one-third of all diabetic patients reporting having had a serious cardiovascular event, including myocardial infarction (MI) or stroke [1]. Those with DM are more likely to have multivessel coronary artery disease, worse outcomes, and increased mortality [2]. Cardiac ischemia is not only a major complication of DKA, but it can also be the cause of ischemia [3]. Unfortunately, the classic symptoms of cardiac ischemia and subsequent MI are unreliable, especially in the setting of DKA [4]. Atypical chest pain is frequently defined as epigastric or back pain, often described as indigestion or recent emesis [5]. Atypical features of ischemia are often overlooked and underestimated, leading to delayed diagnosis and adding to the higher mortality in an already high-risk population. One study demonstrated that the combination of atypical chest pain and a history of DM alone led to delayed admission, delayed treatment, and higher in-hospital death [6].

Radiographic studies in the ED do not play a prominent role in the diagnosis of acute coronary syndrome (ACS). Coronary computerized tomography (CT) angiography has been studied and has the potential to detect large territory infarcts, but this differs significantly from a standard CT scan with intravenous (IV) contrast. To the best of our knowledge, there is minimal literature that demonstrates standard CT identifying ischemic findings of the ACS.

We present a unique case where a CT abdomen and pelvis with IV contrast demonstrated severe hypoattenuation of the posteroinferior aspect of the left ventricular wall, suggesting myocardial infarction in a patient with DKA.

## Case presentation

A 56-year-old female presented to the emergency department with 48 hours of nausea, emesis, generalized abdominal pain, and diarrhea. She initially attributed her symptoms to food poisoning. She denied a specific location of her pain in her abdomen. Her past medical history included hypertension, prior deep vein thrombosis and pulmonary embolism, prior stroke, and insulin-dependent type II DM. She was taking rivaroxaban for her prior thromboembolic disease history. The patient was an everyday smoker and denied alcohol and illicit drug use. At the time of presentation, the patient denied any fever, cough, chest pain, shortness of breath, or recent sick contacts. Upon arrival to the emergency department, the patient was afebrile and hypertensive to 193/83, with otherwise normal vital signs and an unremarkable physical exam, including a nontender abdomen. Initial laboratory studies demonstrated a blood glucose of 392 mg/dL, an anion gap of 18 mEq/L, significant ketones in her urine, and a venous blood gas pH of 7.2. These results were concerning for DKA. The chest X-ray was non-significant. At that time, the patient was given 2 liters of IV lactated ringer crystalloid along with 5 units of insulin lispro prior to being taken for a CT abdomen and pelvis with IV contrast due to continued abdominal pain.

**Figure 1:**
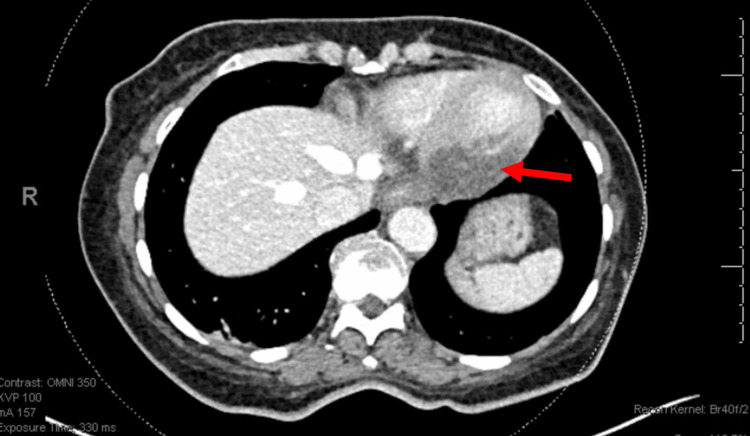
CT scan demonstrating hypoattenuation of the posteroinferior aspect of the left ventricular wall, concerning for acute myocardial infarction.

**Figure 2:**
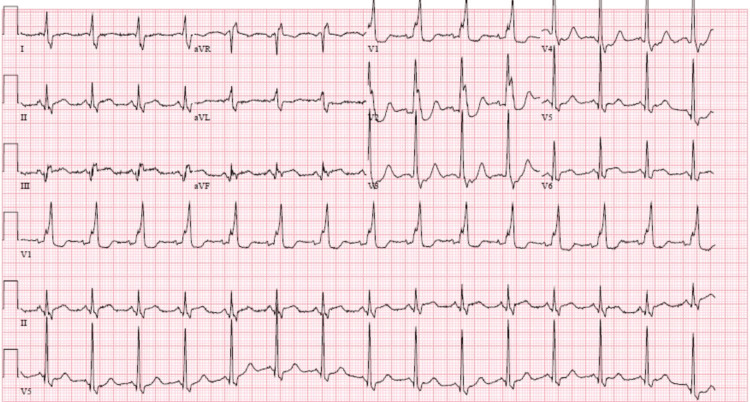
EKG performed after CT scan demonstrating a new right bundle branch block as well as ST segment elevation in leads II, III, aVF, and ST depression in leads V2, V3, V4.

Following the completion of the CT scan (Figure 1), the radiologist called the emergency physician, detailing that the posteroinferior wall of the patient’s left ventricle was not enhancing well, and asked for a clinical correlation. The official radiologist interpretation stated "hypoattenuation of the posteroinferior aspect of the left ventricular wall, which could represent hypoenhancement from acute myocardial infarction or possibly fatty infiltration from prior insult." An EKG was performed after the radiologist's interpretation (Figure 2), revealing ST segment elevations in leads II, III, and aVF, consistent with an inferior STEMI. A STEMI alert was immediately activated, and the cardiology team was notified. Aspirin 325 mg was given, and a heparin drip was initiated. Interventional cardiology took the patient to the catheterization lab. The initial troponin I was found to be 212 ng/mL (nl < 0.04 ng/mL). Interventional cardiology found a 50% stenosed mid-left anterior descending (LAD) segment, a 60% stenosed mid-right coronary artery (RCA) segment, and a mid-circumflex lesion that was 100% stenosed and was likely the culprit lesion. The lesion length was ~10 mm with thrombolysis in myocardial infarction (TIMI) flow of 0. One drug-eluting stent was placed and dilated, resulting in normal blood flow with 0% residual stenosis.

Her troponin would eventually peak at 1152 ng/mL (nl < 0.04 ng/mL), and subsequent transesophageal echocardiography on post-procedure day 1 demonstrated an LV EF of 40-45% with hypokinesis of the basal to mid-inferolateral wall with prolonged relaxation. A review of medical records demonstrated the patient underwent stress echocardiography two years prior, which was significant for no inducible ischemia as well as an ejection fraction of 55% to 60%. The remainder of her hospital course was uncomplicated. Changes were made to her insulin regimen, no other new medications were started, and she was discharged on post-procedure day 2.

## Discussion

This case highlights many important lessons in approaching DM patients who present with DKA. While CT of the abdomen and pelvis with IV contrast should not be routinely performed or relied upon for the diagnosis of MI, radiographic imaging does have a role in critically ill patients with undifferentiated abdominal pain. The literature is replete with cases discussing coronary CT and thoracic CT aortograms in the diagnosis of myocardial ischemia, but the diagnosis of an MI from a contrast-enhanced abdominal and pelvic CT is very rare. Patel et al. described how focusing on myocardial enhancement could allow physicians to detect ischemia, but beyond that, no standardized protocols have been developed [7].

There are many factors that place diabetic patients at higher risk for higher mortality and delayed diagnosis in the setting of ACS [8]. DM and female sex are two independent risk factors for the atypical presentation of MI [6]. Symptoms more suggestive of MI in an atypical presentation include vomiting, abdominal pain, and observed sweating. Patients who present with the metabolic emergency of DKA have similar symptoms of diaphoresis, nausea, emesis, and abdominal pain, which can conceal ACS [9].

Specifically, DM is associated with cardiac autonomic neuropathy (CAN), a condition that greatly influences perceived chest pain [10]. More common in type 2 DM than type 1, CAN result from the activation of inflammatory cytokines leading to abnormalities in sympathetic and vagal regulation [11]. Therefore, the traditional presentation of ischemic chest pain of cardiac origin, described as pressure or tightness, dyspnea, and radiation of symptoms to the neck or arm, is not reliable. While little is known about this condition, some manifestations include resting tachycardia, exercise intolerance, and orthostatic hypotension [9]. Further, it has been found that a patient’s duration of DM is directly correlated to more atypical symptoms during ACS (e.g., difficulty breathing, hyperventilation, indigestion, and unusual fatigue) when compared to patients without diabetes [11].

Providers should have a low threshold for performing screening EKGs on patients with atypical symptoms in the setting of DKA. In this case, the patient was not initially assessed for ACS, and an EKG and troponin level was obtained after CT. Differentiating chest pain from the ischemic origin from other etiologies is difficult, even for skilled providers [8]. Not only early screening EKGs in triage but also serial EKGs during a patient’s ED course greatly assist providers in evaluating for cardiac ischemia. Expectedly, atypical chest pain is associated with delayed presentations for treatment. Delays in diagnosing STEMIs affect long-term patient outcomes, and those with atypical symptoms are at increased risk [12]. The treatment of delayed STEMI patients is associated with significantly more invasive procedures, marked by higher intrahospital mortality and cardiac complications [13].

## Conclusions

This case report highlights a unique instance of radiologic imaging playing a pivotal role in diagnosing myocardial infarction. The case reinforces the variation that is seen in ACS presentations when complicated by diabetes mellitus and DKA. There are many reasons why diabetic patients have atypical chest pain for ACS, and not all are completely understood. Critically, providers must maintain a low threshold to assess for cardiac ischemia in diabetic patients and more readily obtain EKGs in triage as well as during the patient’s course in the ED to prevent complications from delayed ACS care.
